# Phosphorylation Status of Thymidine Kinase 1 Following Antiproliferative Drug Treatment Mediates 3′-Deoxy-3′-[^18^F]-Fluorothymidine Cellular Retention

**DOI:** 10.1371/journal.pone.0101366

**Published:** 2014-07-08

**Authors:** Roberta Sala, Quang-Dé Nguyen, Chirag B. K. Patel, David Mann, Joachim H. G. Steinke, Ramon Vilar, Eric O. Aboagye

**Affiliations:** 1 Comprehensive Cancer Imaging Centre, Department of Surgery and Cancer, Imperial College London, London, United Kingdom; 2 Institute of Chemical Biology, Department of Chemistry, Imperial College London, London, United Kingdom; 3 Department of Life Sciences, Imperial College London, London, United Kingdom; NIH, United States of America

## Abstract

**Background:**

3′-Deoxy-3′-[^18^F]-fluorothymidine ([^18^F]FLT) is being investigated as a Positron Emission Tomography (PET) proliferation biomarker. The mechanism of cellular [^18^F]FLT retention has been assigned primarily to alteration of the strict transcriptionally regulated S-phase expression of thymidine kinase 1 (TK1). This, however, does not explain how anticancer agents acting primarily through G2/M arrest affect [^18^F]FLT uptake. We investigated alternative mechanisms of [^18^F]FLT cellular retention involving post-translational modification of TK1 during mitosis.

**Methods:**

[^18^F]FLT cellular retention was assessed in cell lines having different TK1 expression. Drug-induced phosphorylation of TK1 protein was evaluated by MnCl_2_-phos-tag gel electrophoresis and correlated with [^18^F]FLT cellular retention. We further elaborated the amino acid residues involved in TK1 phosphorylation by transient transfection of FLAG-pCMV2 plasmids encoding wild type or mutant variants of TK1 into TK1 negative cells.

**Results:**

Baseline [^18^F]FLT cellular retention and TK1 protein expression were associated. S-phase and G2/M phase arrest caused greater than two-fold reduction in [^18^F]FLT cellular retention in colon cancer HCT116 cells (p<0.001). G2/M cell cycle arrest increased TK1 phosphorylation as measured by induction of at least one phosphorylated form of the protein on MnCl_2_-phos-tag gels. Changes in [^18^F]FLT cellular retention reflected TK1 phosphorylation and not expression of total protein, in keeping with the impact of phosphorylation on enzyme catalytic activity. Both Ser13 and Ser231 were shown to be involved in the TK1 phosphorylation-modulated [^18^F]FLT cellular retention; although the data suggested involvement of other amino-acid residues.

**Conclusion:**

We have defined a regulatory role of TK1 phosphorylation in mediating [^18^F]FLT cellular retention and hence reporting of antiproliferative activity, with implications especially for drugs that induce a G2/M cell cycle arrest.

## Introduction

Uncontrolled cell proliferation is one of the distinctive features of cancer [1]. Non-invasive imaging of this cancer hallmark can be undertaken with positron emission tomography (PET) [2]. 3′-deoxy-3′-[^18^F]-fluorothymidine ([^18^F]FLT) has been the most widely studied radiotracer [2], [3], [4], [5], [6], [7] for this purpose. Substitution of the 3′-hydroxyl group in [^18^F]FLT by fluorine, confers resistance to catabolism by thymidine phosphorylase [8], [9]. However, due to this substitution, the radiotracer is not efficiently incorporated into the DNA, acting as a chain terminator [2], [10]. Nonetheless, [^18^F]FLT tracks the salvage pathway for DNA synthesis, being efficiently phosphorylated by TK1 [11] and not by TK2 [10], and its uptake in general correlates with measures of conventional cell proliferation markers, such as Proliferating Cell Nuclear Antigen (PCNA), Ki-67, and S phase fraction [2], [7]. The potential of [^18^F]FLT to image tumor proliferation has been reported in several studies [4], [12], [13], [14], and its utility as a pharmacodynamic biomarker to assess efficacy of anticancer therapy has also been evaluated [13], [15], [16].

[^18^F]FLT is transported into cells through facilitated transport *via* the Equilibrative Nucleoside Transporter 1 [17] and phosphorylated by TK1 to produce [^18^F]FLT-monophosphate, which is trapped inside cells ([^18^F]FLTMP) [7], [9], [10], [18]. TK1 is the first enzyme in the salvage pathway [19] and [^18^F]FLT-monophosphate synthesis is rate-limiting for the cellular retention of the nucleoside analogue [20]. In contrast to TK2, TK1 is strictly cell cycle regulated [11], [19]. In actively proliferating cells, TK1 protein expression is reduced in G1 phase, greatly increases (10- to 20-fold) at the G1/S transition, is maintained at high levels throughout S, G2, and M phases (where it reaches maximum levels; [19]) until cell division, before it rapidly decreases at cytokinesis, with the enzyme being degraded at the onset of G1 or G0 [19], [21], [22], [23]. To achieve this oscillation, several regulatory mechanisms are involved, including transcriptional [19], [24] and translational control of expression [21], [25], [26], allosteric [26], [27] and post-translational modifications of the enzyme [23], [28], as well as enzyme degradation [21], [29]. Although TK1 is expressed throughout the cell cycle, its activity is not constant, specifically decreasing at the G2/M transition and during mitosis, when protein expression reaches its peak [23].

To date the mechanism of cellular [^18^F]FLT retention has been assigned primarily to alteration of the strict S phase-regulated expression of TK1. However, this does not account for the impact of some drugs on [^18^F]FLT uptake, particularly those that induce G2/M arrest. In this regard, alternative mechanisms of TK1 regulation during mitosis are hypothesized to be relevant. Chang and co-workers reported TK1 hyperphosphorylation during G2/M phase [23], suggesting that serine-13 (Ser13) was specifically phosphorylated at this stage of the cell cycle [28], reducing TK1 activity and marking the protein for degradation [28], [30]. The same group suggested that TK1 was phosphorylated by a cyclin-dependent kinase (Cdk) - Cdk1 or Cdk2 - although direct evidence is lacking. Cdk1 is a G2/M-specific kinase which regulates events occurring during mitosis after association with specific cyclins; Cdk2 is active during late G1 and S phases, controlling the succession of events during DNA replication [31]. Given the evolving literature on TK1 regulation in mitosis, we investigated post-translational mechanisms of TK1 control involving phosphorylation and its impact on [^18^F]FLT cellular retention during anticancer drug treatment.

## Materials and Methods

### Cell lines and tissue culture

The human colon cancer cell line HCT116 was obtained from ATCC (Manassas, VA) and cultured as described previously [32], [33]. Hos and Ost TK1^−^ human osteosarcoma cells were kindly provided by Prof. Vera Bianchi (Department of Biology, University of Padua, Italy; [34]) and grown in Dulbecco's Modified Eagle's Medium (DMEM) supplemented with 10% fetal bovine serum (FBS), 2 mM L-glutamine and antibiotics (100 U/ml penicillin and 100 µg/ml streptomycin). Cells were maintained in a 5% CO_2_ humidified incubator at 37°C.

### Reagents

Aphidicolin, nocodazole and roscovitine were purchased from Sigma (Gillingham, UK). Paclitaxel was obtained from Calbiochem (Darmstadt, Germany). Calf-intestinal alkaline phosphatase (CIP) was purchased from New England Biolabs (Herts, UK). Lipofectamine 2000 and RNAiMAX transfection reagents were from Invitrogen (Paisley, UK). Phos-tag Acrylamide was initially synthesized by small modifications of a previously reported method (see Protocol S1 for synthetic details) [35], [36] and used to optimize the phospho-TK1 protein bands; subsequently it was purchased commercially from Wako Chemicals GmbH (Neuss, Germany). [^18^F]FLT was purchased from Siemens PETNET Solutions (Nottingham, UK).

### Doubling times assay

50,000 cells were seeded in 6-well plates in triplicates. Cells were trypsinized and counted with the use of a haemocytometer after 24, 48, 72 or 96 hours from seeding.

### Immunoblotting

Cells were lysed in RIPA buffer and proteins denatured in SDS-containing sample buffer. Equal amounts of proteins were subsequently resolved on 12% SDS-PAGE and transferred on polyvinylidene fluoride membranes. For MnCl_2_-phos-tag gels, 75 µM phos-tag and 150 µM MnCl_2_ were added to 12% resolving gel (12% acrylamide, 375 mM Tris, 0.1% SDS, pH 8.8). Before electrotransfer, gels were washed 3 times in 0.5 M EDTA pH 8.0 to remove metal complexes. Blots were probed with specific primary antibodies, and proteins of interest were visualized *via* enhanced chemiluminescence after incubation with horseradish peroxidase (HRP)-conjugated secondary antibodies. Mouse anti-thymidine kinase 1 and mouse anti-thymidylate synthase antibodies were purchased from Abcam (Cambridge, UK). Rabbit anti-Cdk1 and anti-Cdk2 were obtained from Cell Signaling Technology (Herts, UK). Rabbit anti-actin antibody was purchased from Sigma. Anti-mouse and anti-rabbit HRP-conjugated antibodies were from Santa Cruz Biotechnology (Heidelberg, Germany).

### DNA cell cycle analysis

Cell cycle distribution was assessed using propidium iodide (PI, Sigma) and DNA-based flow cytometric analysis in fixed, permeabilized cells, as previously described [37]. Cells were analyzed in a BD FACS Canto cytometer (BD, Oxford, UK), counting 10,000 cells per sample. Selective gating was applied to exclude cell debris and doublets from the analysis. Cell cycle distribution was analyzed with FlowJo version 7.6.5 (Tree Star, Ashland, USA).

### Transient transfections of Ost TK1^−^ cells

FLAG-pCMV2 plasmids encoding for Δmet wild type TK1, Δmet S13A TK1, Δmet S13D TK1 or Δmet S231A TK1 were kindly donated by Dr. Chang, University of Taiwan. Adherent cells were incubated with Lipofectamine 2000 transfection reagent and the selected plasmid, according to the manufacturer's instructions. The transfection mixture was removed 6 hours post transfection and replaced with fresh media, containing nocodazole as required. Transgene expression was tested 24 hours after transfection *via* immunoblot analysis.

### RNA interference

Human Cdk1 and Cdk2 ON-TARGETplus SMARTpool siRNA and ON-TARGETplus non-targeting pool (scrambled) were purchased from Dharmacon (Dharmacon Lafayette, CO, USA). HCT116 cells were transfected with Cdk1, Cdk2 or scrambled siRNA for 48 hours using wet reverse transfection with RNAiMAX transfection reagent, according to manufacturer's instructions. Validation of knockdown was done by immunoblot and qPCR.

### [^18^F]FLT cell uptake

Cells were seeded in 6-well plates in triplicates to achieve ∼60% confluency, and treated with cell-cycle arresting agents 24 h prior the experiment. About 0.37 MBq (10 µCi) of radioactivity was added to each well and incubated for 1 h in a 5% CO_2_ humidified incubator at 37°C. Cells were subsequently scraped in media, and spun at 6,500 rpm at 4°C for 2 min. Cell pellets were washed twice with PBS, before being resuspended in 200 µl RIPA buffer, transferred into counting tubes and analyzed for radioactivity counts on a Cobra II Auto-Gamma counter (Packard Biosciences Co., Pangbourne, UK).

### Statistical analysis

Data were analyzed using GraphPad Prism, version 5 (San Diego, USA), applying the Student's two-tailed t-test to calculate statistically significant differences between data sets, and are represented as mean ± Standard Error of the Mean (S.E.M.). Significant differences were expressed as * (p<0.05), ** (p<0.01) or *** (p<0.001).

## Results

### Baseline TK1 total protein expression determines [^18^F]FLT uptake but not proliferation

**Figure 1:**
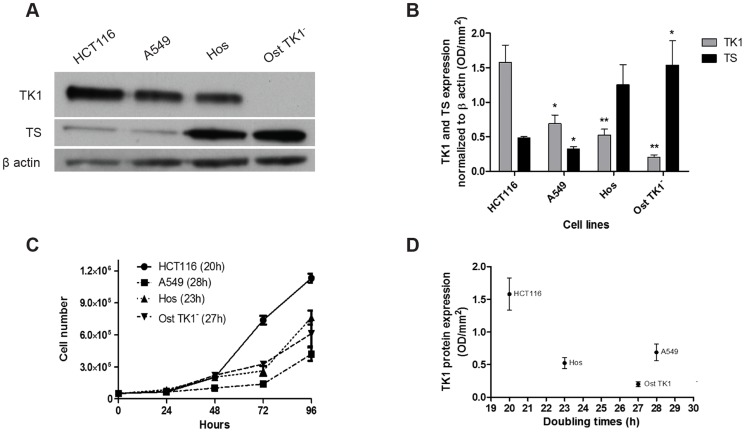
Relationship between thymidine kinase 1 (TK1) and thymidylate synthase (TS) expression, and doubling time. A) Representative western blot showing TK1, TS and β actin expression in the analyzed cell lines. Cells were grown in 10 cm plates for 48 h to reach approximately 80% confluency at the time of the analysis. B) Quantification of TK1 and TS expression normalized to β actin. Average of 3 independent experiments. HCT116 are used as reference to calculate statistical significance. C) Cell proliferation rates of HCT116, A549, Hos and Ost TK1^−^ cells. Cells were seeded in 6-well plates in triplicates. Doubling times (DT) were calculated using the macro available on http://www.doubling-time.com/compute.php?lang=en. D) Representative correlation plot between doubling times of each cell line and TK1 protein expression.

**Figure 2:**
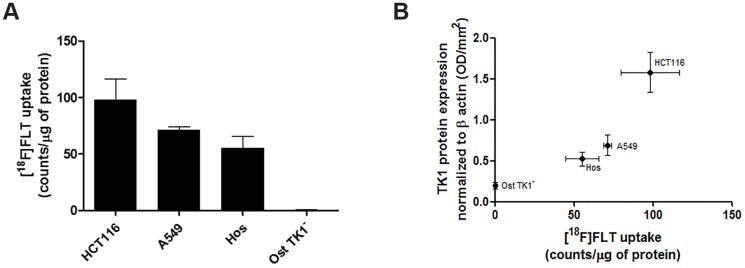
Comparison of thymidine kinase 1 (TK1) expression to [^18^F]FLT uptake. A) [^18^F]FLT uptake in HCT116, A549, Hos and Ost TK1^−^ cells. B) Plot showing the association between [^18^F]FLT uptake and TK1 protein expression. Values are means ± S.E.M.

TK1 expression was investigated in four cell lines (Figure 1A and B), together with thymidylate synthase (TS) expression to account for possible differences in the usage of the salvage versus the *de novo* pathway for thymidine production. Cells lacking TK1 (Ost TK1^−^) showed high TS expression signifying an ability to synthesize DNA *via* the *de novo* pathway. For cells that were proficient for TK1, TS expression ranged from low to high (Figure 1A and B) indicating that either pathway could be used depending on available substrates. The shortest doubling time was found in HCT116 cells that had the highest TK1 expression; given that only 4 cell lines were assessed we did not assign quantitative biological significance to the association between doubling time and TK1 expression (Figure 1C and D). As [^18^F]FLT cellular uptake reflects TK1 activity [7], [18], we found undetectable radiotracer uptake in Ost TK1^−^ cells lacking the protein (Figure 2A, 2B). [^18^F]FLT uptake was highest in HCT116 cells that had the highest TK1 expression of the four cell lines (Figure 2A, 2B), in keeping with a role for TK1 in mediating the extent of usage of the salvage pathway and hence [^18^F]FLT uptake.

### TK1 phosphorylation during cell-cycle progression can determine [^18^F]FLT uptake independent of expression

**Figure 3:**
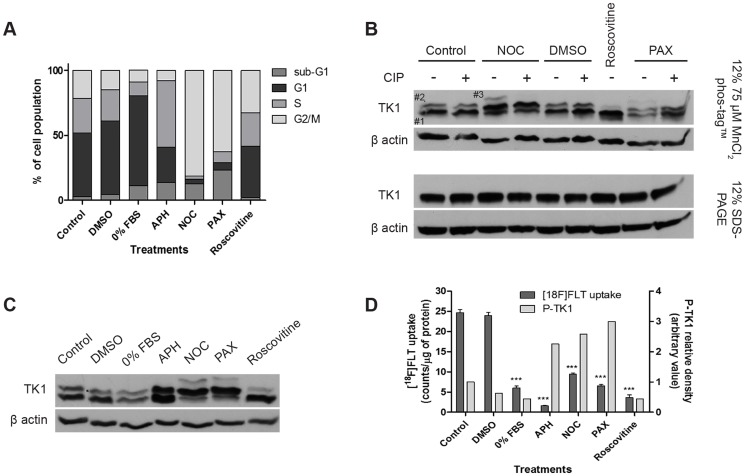
Effect of drug-induced alteration of cell cycle progression on TK1 phosphorylation and [^18^F]FLT cell uptake. The following drug concentrations were used in each experiment: 10 µM aphidicolin (APH), 0.5 µg/ml nocodazole (NOC), 250 nM paclitaxel (PAX), 50 µM roscovitine. A) Representative cell cycle distribution profiles of HCT116 cells after 24 h drug-induced cell cycle arrest. Treatment with 50 µM roscovitine was carried out for 4 h. B) Characterization of TK1 phosphorylation bands (using 75 µM MnCl_2_-phos-tag gels) in cell cycle arrested HCT116 cell lysates by alkaline phosphatase (CIP) treatment (100 U per 100 µg of protein at 37°C for 1 h). HCT116 cells were treated with nocodazole or paclitaxel for 24 h. Roscovitine treatment was carried out for 4 h. C) Representative TK1 phosphorylation profile after treatment with the indicated agents for 24 h (4 h for roscovitine treatment). D) [^18^F]FLT uptake and phospho-TK1 (P-TK1) expression in HCT116 following treatments for 24 h (4 h for roscovitine treatment). The left y-axis represents mean ± S.E.M. expressed as quantitative counts per µg of protein. Stars highlight significant differences compared to untreated control. The right y-axis represents semiquantitative P-TK1 density (band #2+band #3). An arbitrary value of 1 was assigned to P-TK1 expression in control cells and this value was used as comparison for the other treatments.

We hypothesized that TK1 would be subject to phosphorylation events during progression through the cell cycle [23], [28], [38], with potential impact on [^18^F]FLT cellular retention. A number of agents were used to modulate the cell cycle in order to assess the effect of phosphorylation on [^18^F]FLT cellular retention. These included serum starvation (0% FBS; G0/G1 arrest), treatment with the DNA polymerase inhibitor aphidicolin (S-phase arrest), microtubule interfering agents nocodazole and paclitaxel (G2/M arrest), and roscovitine (non-specific CDK inhibition; active in G1 and M-phase). The effect of these agents on the cell cycle – compared to vehicle (DMSO) or untreated controls - was confirmed by flow cytometry in HCT116 cells (Figure 3A). Approximately 70% G1, 51% S phase, and 77%/61% G2/M cells were observed with 0% FBS, aphidicolin and nocodazole/paclitaxel, respectively. Treatment with 50 µM roscovitine for 4 hours was unremarkable (2.25% sub-G1, 39.2% G1, 25.5% S, 32.5% G2/M).

Phosphorylated and unphosphorylated TK1 proteins were separated on 12% SDS-PAGE containing 75 µM MnCl_2_-phos-tag. This gel system allows the separation of phosphorylated forms of a protein due to phosphate groups interacting with the MnCl_2_-phos-tag, which is therefore responsible for inducing a mobility shift of the phosphorylated species [39]. To confirm that the assay indeed measures phosphorylated proteins, cells treated with a subset of the agents above were processed with or without alkaline phosphatase (CIP) to dephosphorylate TK1 prior to gel electrophoresis. While use of 12% SDS-PAGE alone produced a single band corresponding to the TK1 protein, samples separated on 12% SDS-PAGE containing 75 µM MnCl_2_-phos-tag showed 3 discernible bands. Band #3 was dephosphorylated by CIP, thus, confirming identity as a phospho-protein (Figure 3B). As positive control, CIP dephosphorylation was also demonstrated using phospho-AKT antibody (Figure S1). Band #2 was not dephosphorylated by CIP, possibly due to inaccessibility of CIP to the phosphorylated site, however, treatment with roscovitine for 4 hours reduced the signal from this band, suggesting that band #2 could be a phospho-protein (Figure 3B). Further studies with mutant TK1 (S13A; *vide infra*) also supported this assertion.

TK1 phosphorylation was then assessed against the full set of cell cycle modulating conditions in unsynchronized cells (confirmed in synchronized cells –Figure S2). G0/G1 arrest led to a reduction of total TK1 expression (bands #1 and #2; Figure 3C) while S-phase arrest increased its expression. G2/M arrest induced by two different drugs, led to a decrease in band #1 and increase in band #2, as well as induction of the G2/M specific band #3. TK1 phospho-protein is reported to have a reduced propensity to form the tetrameric form of TK1 with high enzymatic activity [26], [28], [38]. Roscovitine led to a lower expression of band #2, a putative phospho-protein. The data presented above indicate that in HCT116 cells, a phosphorylated form of TK1 is present in all phases of the cell cycle while additional protein forms are induced in M-phase. Not surprisingly, the G0/G1 arrest-induced reduction in total TK1 expression led to 5-fold lower [^18^F]FLT cellular retention (p<0.0001) compared to control (Figure 3D). During G1 phase, thymidine is not needed and therefore TK1 protein expression and activity are down-regulated [40]. The impact of other conditions was unexpected. S-phase arrest while leading to higher expression of total protein also led to lower [^18^F]FLT cellular retention by 90% (p<0.0001) suggesting that the protein formed was likely less active (mixture of non-phosphorylated and phosphorylated proteins). This finding is perhaps also explained by the negative feedback generated by thymidine triphosphate (dTTP) accumulation during S-phase arrest; dTTP is not incorporated into the DNA due to inhibition of DNA polymerase, therefore it interacts with TK1 to modulate its activity [27], [41], [42], preventing nucleotide imbalance which could cause genetic instability [26]. Furthermore, M-phase arrested cells that had increased band #2 together with an additional phospho-protein band (band #3) also showed lower [^18^F]FLT cellular retention (p<0.0001) similarly indicating inactive protein. This is in line with the notion that at the end of DNA replication, TK1 enzymatic activity is no longer needed for the production of thymidine monophosphate, and is therefore down-regulated [28]. Roscovitine also led to a lower [^18^F]FLT cellular retention (p<0.0001), in spite of its unremarkable cell cycle arrest profile (Figure 3A), but consistent with a reduction in phospho-band #2 (and therefore total protein) *via* inhibition of CDK activity.

### Identification of the phosphorylated amino-acid residues on TK1 protein responsible for modulation of [^18^F]FLT cellular retention

**Figure 4:**
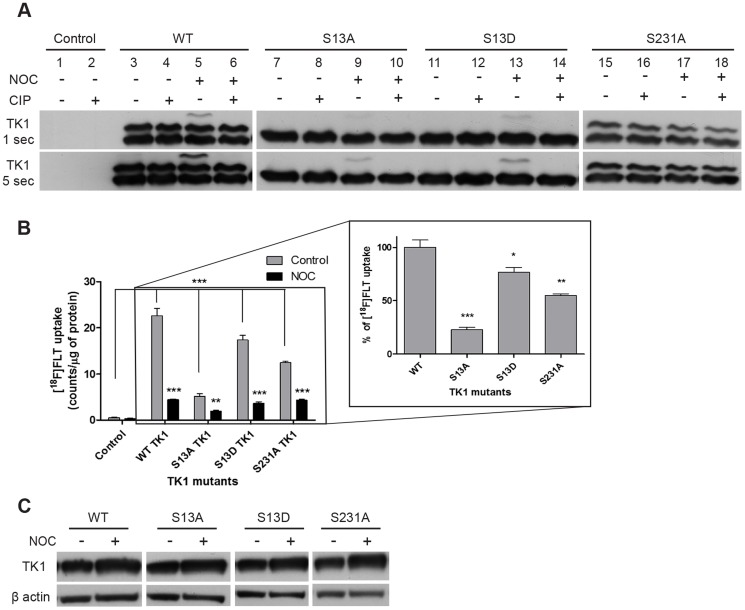
Verification of phosphorylated amino acid residues on TK1 protein. Ost TK1^−^ cells were transiently transfected with FLAG-pCMV2 plasmids coding for TK1 variants. A) Ost TK1^−^ transient transfections with FLAG-pCMV2 plasmids coding for the indicated TK1 variants were resolved (10 µg) on a 75 µM MnCl_2_-phos-tag gels and probed for TK1 expression (1∶500). B) [^18^F]FLT uptake in Ost TK1^−^ cells transiently transfected with the indicated TK1 constructs. The main figure represents the uptake in control and nocodazole (0.5 µg/ml) treated samples. The y-axis represents mean ± S.E.M. expressed as counts per µg of protein. Significant differences following transient transfection compared to non-transfected cells, and significant changes after nocodazole treatment compared to the untreated sample are highlighted by stars. The inset reports the percentage of radiotracer uptake compared to cells transfected with wild type (WT; 100%) TK1. Significant differences are indicated by stars. C) TK1 expression in transfected cells resolved on 12% SDS-PAGE. β actin was used as loading control.

In order to verify the differential phosphorylations on TK1 protein, Ost TK1^−^ cells were transiently transfected with FLAG-pCMV2 plasmid vectors coding for TK1 containing different point mutations. TK1 phosphorylation at two amino acid residues – position 13 and 231 – were studied; mutation of Serine-13 (Ser13) and Ser231 to alanine (S13A and S231A, respectively) was intended to eliminate phosphorylation, while mutation of Ser13 to aspartate (S13D) was intended to mimic phosphorylation. Transfection with wild-type TK1 (WT; Figure 4A, lanes, 3–6) produced the expected profile observed in cells expressing endogenous TK1, with 2 bands (band #1 and #2) present under each condition and an extra band (band #3) upon nocodazole-induced G2/M arrest. Band #3 was abolished after dephosphorylation by CIP. Substitution of Ser13 with alanine (S13A; Figure 4A, lanes 7–10) or aspartate (S13D; Figure 4A, lanes 11–14) reduced the TK1 profile to a single band (band #1). An additional signal (band #3) appeared upon G2/M arrest, albeit less intense, and was abolished after dephosphorylation by CIP. In contrast, when Ser231 was substituted by alanine (S231A), bands #1 and #2 were observed; however, band #3 was not induced when similarly treated with nocodazole. It should be noted that band intensities for the S231A panel was fainter than for WT TK1 even with longer exposure of the blots. Thus, Ser13 substitution abolished band #2 (which is a phospho-protein band that is constitutively expressed with further induction by M-phase arrest, or a mixture of phosphorylated species including that which is constitutively expressed throughout the cell cycle and a M-phase arrest inducible phospho-protein) while S231A abolished band #3. It is therefore reasonable to conclude that band #1 is the non-phosphorylated protein; phosphorylation of Ser13 contributes to band #2; and phosphorylation of Ser231 contributes to band #3.

### Determining the role of TK1 differential phosphorylation on enzymatic activity

Identifying the functional consequence of phosphorylation on different amino acid residues was deemed important for characterizing the oscillation of TK1 activity during cell-cycle progression. For this reason, [^18^F]FLT cellular retention, which in a single cell line model (with identical cellular transport kinetics) is a direct measure of TK1 enzymatic activity, was performed to determine if the specific phosphorylation profiles were linked to differential enzymatic activity. As shown in Figure 4B, each transfection produced significantly higher [^18^F]FLT cellular retention in Ost TK1^−^ cells transfected with TK1 vectors (p = 0.0002 for WT, p = 0.0009 for S13A, p<0.0001 for both S13D and S231A) compared to untransfected Ost TK1^−^ cells. For each transfection, treatment with nocodazole for 24 hours led to lower [^18^F]FLT cellular retention compared to the corresponding vehicle-treated samples (p<0.005; 5-fold for WT, 2 fold for S13A and 4-fold for S13D). Finally, nocodazole treatment of cells expressing S231A mutated TK1 caused a 3-fold lower [^18^F]FLT cellular retention compared to the vehicle treated S231A cells. Figure 4B (inset) represents the percentage of radiotracer uptake relative to cells transfected with WT TK1, assigning an arbitrary value of 100% to the uptake values measured in this sample. Eliminating phosphorylation *via* S13A substitution was responsible for an 80% lower [^18^F]FLT cellular retention compared to WT (p = 0.0005), whereas mimicking TK1 phosphorylation (S13D mutation) showed only a 20% lower (p<0.05) cellular retention. Eliminating phosphorylation *via* S231A resulted in lower radiotracer retention, although not as marked as with S13A, being 50% of the WT retention value (p = 0.0033). Figure 4C shows similar expression of TK1 following transfection with TK1 constructs in Ost TK1^−^ cells, indicating that the effects on [^18^F]FLT cellular retention were not caused by differences in protein expression between the TK1 variants, but were generated by the specific mutations.

Taken together, the results presented here suggests that Ser13 phosphorylation might be important for activating the TK1 enzyme, since compared to WT, [^18^F]FLT uptake was reduced after substitution with alanine; this is in contrast to the previously described effect of Ser13 substitution on TK1 activity during G2/M [28]. Radiotracer uptake was further decreased after nocodazole treatment of cells expressing TK1 with the S13A mutation, indicating that Ser13 may be one of a number of amino acid residues phosphorylated during the G2/M phase. Results obtained with the S13D mutant that mimics phosphorylation of TK1 (Asp13 mimics the change of charge induced by phosphorylation at the same position [30], [38]), further corroborated our speculation, since substitution of Ser13 with Asp13 only minimally lowered [^18^F]FLT cellular retention. This result is in contrast with the report by Li *et al.* [38], which concluded that the phosphorylation on Ser13, mimicked by the S13D substitution, was responsible for decreased affinity for the substrate due to dissociation of the tetrameric form of TK1 to form the dimeric form. However, a 20% decrease in radiotracer uptake is still notable, indicating that a change in TK1 affinity for the substrate might have occurred following S13D substitution. Finally, the results obtained with the Ser231 substitution suggested that: 1) S231A mutation has an impact on TK1 enzymatic activity due to the 50% decrease in [^18^F]FLT uptake, and 2) there might be residues, different from the ones investigated in this study, which are phosphorylated on TK1 protein; in fact, there was still a decrease in [^18^F]FLT uptake after nocodazole treatment, in contrast to the expected result, i.e., no difference between nocodazole treated and non-treated S231A samples.

### Which kinase is responsible for TK1 phosphorylation?

**Figure 5:**
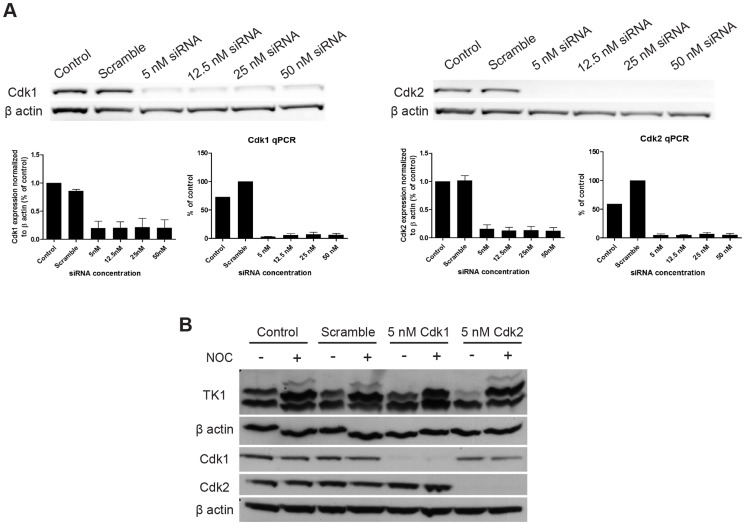
Determination of the regulation of TK1 phosphorylation by Cdk1 and Cdk2. A) Cdk1 and Cdk2 knock-down optimization. Representative western blots, with corresponding densitometry (average of 3 independent experiments) and qPCR analysis. Control refers to non-silenced cells; scramble indicates transfection with non-targeting siRNA. B) Determination of the Cdk responsible for TK1 specific phosphorylation. 5 nM of scramble, Cdk1 or Cdk2 siRNA were used to induce Cdk knock-down for 48 h. Cells were treated O/N with 0.5 µg/ml nocodazole (NOC) following Cdk knock-down. Top two panels represent MnCl_2_-phos-tag gel; bottom three panels report 12% SDS-PAGE.

It has been suggested that TK1 is phosphorylated in G2/M by Cdk2 or Cdk1 [28], but to the best of our knowledge no direct evidence for such effect has been reported. We attempted to identify which of these two CDKs was involved in phosphorylation of the bands corresponding to the different amino acid residues. Cdk1 or Cdk2 was knocked-down by RNA interference (RNAi) in HCT116 cells. Knock-down was confirmed even at the lowest siRNA concentration used (5 nM; Figure 5A), with at least a 70% reduction in mRNA expression. After 48 hours knock-down with 5 nM siRNA against either Cdk1 or Cdk2 and overnight treatment with nocodazole, HCT116 whole cell lysates were run on 12% SDS-PAGE containing 75 µM MnCl_2_-phos-tag to probe for TK1 phosphorylation, as well as on 12% SDS-PAGE to test for effective Cdk1 and Cdk2 protein knock-down (Figure 5B). As expected, G2/M specific phosphorylation was induced after nocodazole treatment in both control and non-targeting siRNA-treated samples (Figure 5B, lanes 2 and 4, respectively), with band #2 increasing at the same time. Knock-down of Cdk1 resulted in the absence of the G2/M specific nocodazole-induced phosphorylation (band #3; Figure 5B, lane 6). The Cdk2 knockdown matched more closely what was observed with roscovitine (Figure 3B). In fact, Cdk2 knock-down seemed to have an effect on phosphorylation of band #2 only in asynchronized cells (no nocodazole treatment), with the signal coming from this form increasing after induction of G2/M arrest. This effect could be explained hypothesizing that additional CDKs might have substituted Cdk2 in its role of phosphorylating specific substrates [43], [44] or that additional residues (which could not be separated on phos- tag gels) were phosphorylated during G2/M. Further validation is required to confirm these results.

## Discussion

We demonstrated that drug induced G2/M arrest leads to a reduction in [^18^F]FLT cellular retention that is reflected in TK1 phosphorylation status rather than total protein expression. The mechanism of cellular [^18^F]FLT retention has been assigned primarily to alteration of the strict transcriptionally regulated S-phase expression of TK1 [7], [45]. This, however, does not explain how anticancer agents acting primarily through G2/M arrest affect [^18^F]FLT uptake. Recent reports show that indeed, drugs that classically induce G2/M arrest also cause a reduction in [^18^F]FLT uptake [46] but the mechanism is unclear. We investigated alternative mechanisms of [^18^F]FLT cellular retention involving post-translational modification of TK1 during mitosis. Cells are able to use both the *de novo* and salvage pathways for synthesizing DNA nucleosides precursors. Even though this property together with levels of thymidine can skew the relationship between [^18^F]FLT uptake and TK1 protein expression [47], [48], we show here in four cell lines with varying TK1 expression a linear relationship between TK1 and [^18^F]FLT cellular retention, similar to other previous reports [7], [18], [49].

Our hypothesis that G2/M arrest can alter [^18^F]FLT cellular retention is inspired by the work of Chang and co-workers [28] who noted that G2/M arrest led to enhanced phosphorylation of TK1 on Ser13. Following this lead, we were able to separate phosphorylated forms of TK1 by a charge-dependent electrophoresis method. We show that TK1 phosphorylation by drugs that induce G2/M arrest leads to a reduction in [^18^F]FLT. This was not related to reduction in protein expression but rather protein activity. While the focus is on G2/M, it should be noted that S-phase arrested cells also showed increased total TK1 expression and differential phosphorylation. These findings suggest caution against the use of total TK1 expression, e.g., in the form of immunohistochemistry, for monitoring the effect of drugs that induce G2/M (or indeed S-phase) arrest. The situation is different for drugs affecting the Rb-E2F axis in G1 phase of the cell cycle, and hence TK1 transcription [50], [51].

The specific amino acid residues involved in TK1 phosphorylation-modulated [^18^F]FLT cellular retention are also of interest. We approached identification of phosphorylated amino acid residues by transfecting TK1-deficient cells (Ost TK1^−^) with DNA plasmid vectors encoding mutant variants of TK1 cDNA. A previous report [28], identified Ser13 phosphorylation during G2/M as responsible for TK1 reduced activity at this stage of the cell cycle, causing dissociation of the more active tetrameric form of the TK1 protein. Our findings, however, suggested that Ser13 might be required for TK1 enzymatic activity. This assertion is evidenced by the fact that substitution with an alanine, which precludes phosphorylation, did not maintain [^18^F]FLT uptake, i.e. TK1 activity, close to that of wild type TK1 protein, even after nocodazole treatment, assuming that Ser13 was specifically phosphorylated during G2/M. On the contrary, S13A substitution caused a significant decrease in [^18^F]FLT uptake compared to control, suggesting a role in increasing the activity of the enzyme. Moreover, nocodazole treatment of cells transfected with the S13A variant of TK1 showed a significant decrease in radiotracer retention compared to untreated samples, suggesting that the phosphorylation of a different residue might in part be responsible for the TK1 activity reduction. Ser231 appeared to be an important residue phosphorylated during mitosis in our studies; this finding is supported by the identification of TK1 phosphorylation on Ser231 in proteomic studies [52], [53]. Again, it might not be the only phosphorylated residue during G2/M phase, as evident from [^18^F]FLT cell uptake studies following nocodazole treatment. In this regard, other amino acid residues on TK1 including Ser30 and Ser194 have been suggested to play a role in G2/M arrest [28], and thus need to be characterized. Putative kinases involved in TK1 phosphorylation were also investigated, concentrating on Cdk1 and Cdk2. We showed through silencing experiments that Cdk1 is the likely candidate to phosphorylate TK1 during G2/M with the G2/M specific phosphorylation being abolished after Cdk1 knock-down, but visible in nocodazole-treated Cdk2 knock-down cells. We could not however rule out an effect by Cdk2.

In conclusion, we have defined a regulatory role of TK1 phosphorylation in mediating [^18^F]FLT cellular retention and hence reporting of antiproliferative activity, with implications especially for drugs that induce G2/M cell cycle block. We demonstrated the presence of at least two phosphorylated residues on TK1 sequence, which are responsible for modulating the activity of the enzyme in relation to [^18^F]FLT cellular retention. Indeed, changes in [^18^F]FLT cellular retention following G2/M-arresting drug treatment reflected TK1 phosphorylation and not expression of total protein, in keeping with the impact of phosphorylation on enzyme catalytic activity when DNA synthesis has already terminated [8], [54]. The studies presented here support the use of [^18^F]FLT PET for evaluating treatment of patients with drugs that inhibit G2/M including taxanes [45], as well as underpin development of novel G2/M inhibitors.

## Supporting Information

Figure S1 **Validation of Akt dephosphorylation as a control for protein dephosphorylation by CIP.** HCT116 cells were grown in complete medium (10% FBS) or serum-deprived medium (0% FBS) for 24 h. Insulin treatment was performed for 1 h to enhance AKT protein phosphorylation, after which cells were lysed and incubated with CIP for 1 h.
(TIF)

Figure S2 **TK1 phosphorylation profile in synchronized HCT116 cells.** HCT116 cells were serum starved for 24 h, and subsequently incubated in fresh medium, containing 10% FBS and 0.5 µg/ml nocodazole (NOC) or 250 nM paclitaxel (PAX) as required, for 18 h (O/N), 24 h or 48 h. Cells were lysed for western blotting or analyzed *via* flow cytometry. A) Representative resolution of TK1 phosphorylated isoforms on 12% acrylamide+75 µM MnCl_2_-phos-tag after cell synchronization. B) Representative cell cycle analysis of DNA content measured after propidium iodiode (PI) staining. O/N = overnight; NT = non-treated; NOC = nocodazole; PAX = paclitaxel.
(TIF)

Protocol S1 **Synthetic details for the preparation of phos-tag acrylamide.**
(DOCX)
