# Supplementary figures and images for: Phosphorylation Status of Thymidine Kinase 1 Following Antiproliferative Drug Treatment Mediates 3′-Deoxy-3′-[18F]-Fluorothymidine Cellular Retention
Source: PLoS One. 2014 Jul 8;9(7):e101366. doi: 10.1371/journal.pone.0101366 (PMC4086825; doi:10.1371/journal.pone.0101366)

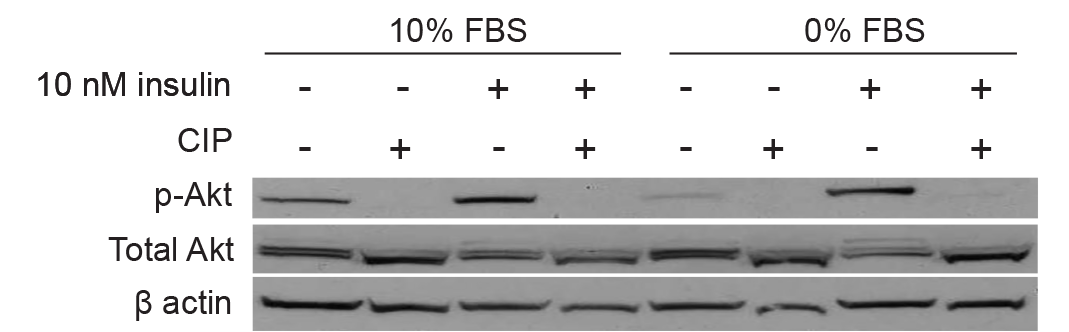

Supplement: Figure S1 — Validation of Akt dephosphorylation as a control for protein dephosphorylation by CIP. HCT116 cells were grown in complete medium (10% FBS) or serum-deprived medium (0% FBS) for 24 h. Insulin treatment was performed for 1 h to enhance AKT protein phosphorylation, after which cells were lysed and incubated with CIP for 1 h. (TIF) [file pone.0101366.s001.tif]

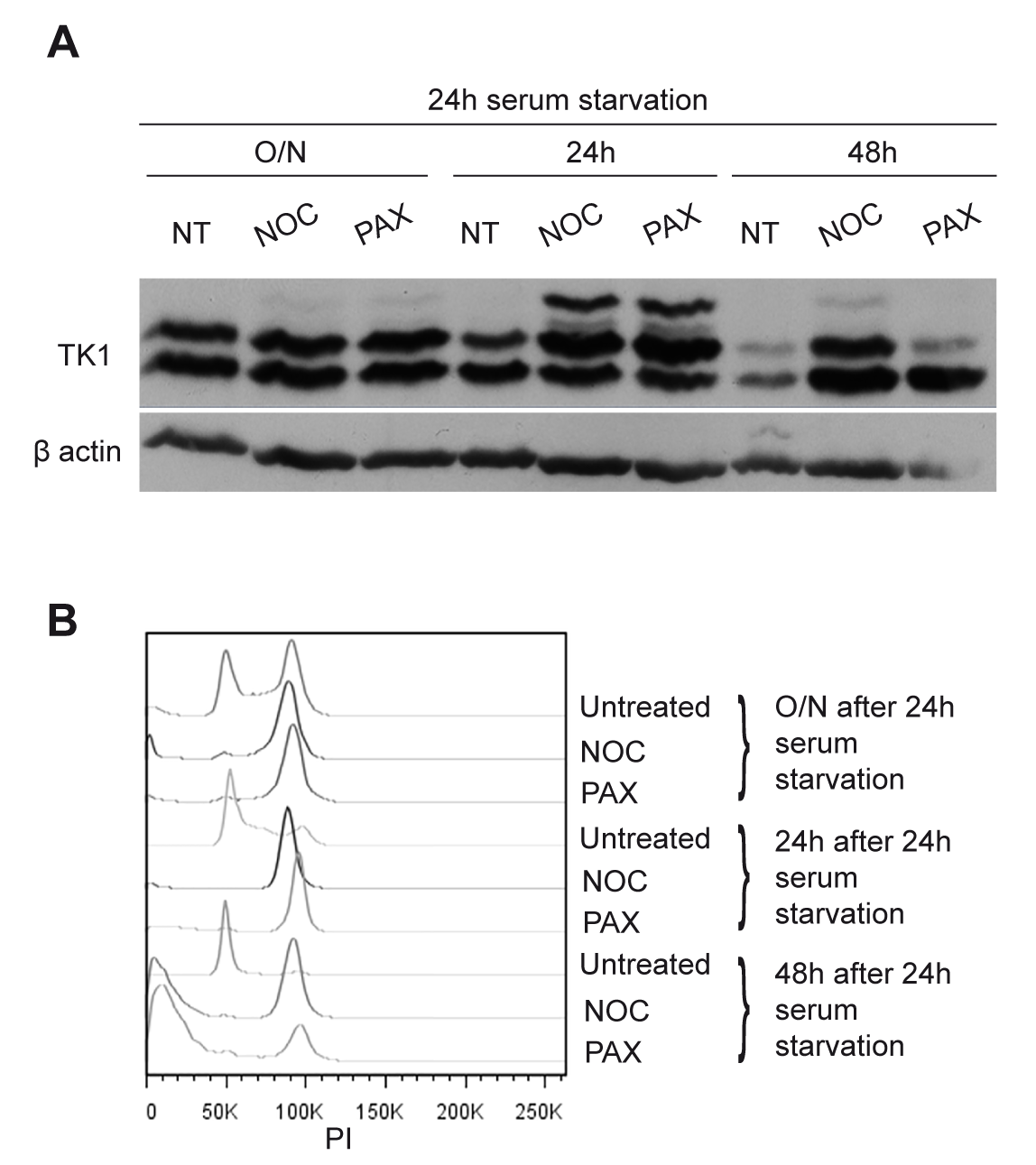

Supplement: Figure S2 — TK1 phosphorylation profile in synchronized HCT116 cells. HCT116 cells were serum starved for 24 h, and subsequently incubated in fresh medium, containing 10% FBS and 0.5 µg/ml nocodazole (NOC) or 250 nM paclitaxel (PAX) as required, for 18 h (O/N), 24 h or 48 h. Cells were lysed for western blotting or analyzed via flow cytometry. A) Representative resolution of TK1 phosphorylated isoforms on 12% acrylamide+75 µM MnCl2-phos-tag after cell synchronization. B) Representative cell cycle analysis of DNA content measured after propidium iodiode (PI) staining. O/N = overnight; NT = non-treated; NOC = nocodazole; PAX = paclitaxel. (TIF) [file pone.0101366.s002.tif]
