# Supplementary material for: Phosphorylation Status of Thymidine Kinase 1 Following Antiproliferative Drug Treatment Mediates 3′-Deoxy-3′-[18F]-Fluorothymidine Cellular Retention
Source: PLoS One. 2014 Jul 8;9(7):e101366. doi: 10.1371/journal.pone.0101366 (PMC4086825; doi:10.1371/journal.pone.0101366)
Supplement: Protocol S1 — Synthetic details for the preparation of phos-tag acrylamide. (DOCX) [file pone.0101366.s003.docx]

**Supplementary Methods**

**Protocol S1. Synthetic details for the preparation of Phos-Tag^TM^ acrylamide.**

The synthesis of Phos-Tag™ acrylamide **6** was successfully achieved via 6 steps (see Scheme S1) with all precursor identified by spectroscopic analysis with overall yield of 9 %.

|  |
| --- |

**Scheme S1.** Synthesis of *N*-(2-acrylamidoethyl)-2-(((3-(bis(pyridin-2-ylmethyl)amino)-2-hydroxypropyl)(pyridin-2-ylmethyl)amino)methyl)isonicotinamide

**1-(bis(pyridin-2-ylmethyl)amino)-3-(pyridin-2-ylmethylamino)propan-2-ol (1).** This compound was prepared by slight modifications of a previously reported procedure.^2^ To a solution of 1,2-diaminopropan-2-ol (1.08 g, 12.1 mmol) and conc. hydrochloric acid (2 mL) in methanol (80 mL), was added 2-pyridine aldehyde (3.88 g, 36.3 mmol) dropwise resulting in a dark orange solution. Sodium cyanoborohydride (1.67 g, 26.6 mmol) was added to the solution in small portions turning the solution opaque yellow. After the addition was completed, the mixture was stirred at room temperature for 72 h. The resulting solution was made acidic by adding conc. hydrochloric acid until the pH was 1. The solution was then adjusted to pH 8 by the addition of 0.1 M sodium hydroxide, followed by extraction by chloroform (3 × 50 mL). The extracts were then collected and dried over sodium sulphate and concentrated under reduced pressure. The residue obtained was then purified by silica gel column chromatography (dichloromethane/methanol/NH_3_(aq., 28% w/w) v/v/v 50:10:1→20:10:1), thereby yielding amine **1** (1.36 g, 31%) as a dark yellow/orange oil. R*_f_* 0.22, (dichloromethane/methanol/NH_3_(aq., 28% w/w) v/v/v 50:10:1); ^1^H NMR: δ_H_ (CDCl_3,_ 400MH_z_) 2.71 (m, 4H, CHOHC*H_2_*), 3.96 (m, 7H, NC*H_2_*, C*H*OH), 7.15 (m, 3H, py 5-*H*), 7.36 (m, 3H, py 3-*H*), 7.61 (m, 3H, py 4-*H*) and 8.53 (m, 3H, py 6-*H*); ^13^C NMR: δ_C_ (CDCl_3,_ 100.7MH_z_) 53.2, 55.0, 59.4, 60.5, 67.7, 122.1, 122.1, 122.3, 123.1, 136.6, 149.0, 149.2, 159.1 and 159.2; MS (ESI^+^) *m/z* 364 (MH^+^, 100); HRMS (ESI^+^) calcd for C_21_H_26_N_5_O (MH^+^) 364.2139, found 364.2129 (Δ = - 2.5 ppm). Data consistent with literature.^2^

**Methyl 6-(hydroxymethyl)nicotinate (2).** This compound was prepared by slight modifications of a previously reported procedure.^5^ To a slurry of pyridine 2,5-dicarboxylate (308 mg, 1.58 mmol) and calcium chloride (693 mg, 6.25 mmol) in tetrahydrofuran (3.3 mL) and methanol (6.7 mL) was added sodium borohydride (149 mg, 3.95 mmol) at -78˚C in small portions. The resulting mixture was warmed slowly to 0˚C over 5 h, poured into ice/water (5 mL), and extracted with chloroform (3 × 20 mL). The extracts were then collected and dried over Na_2_SO_4_ and concentrated under reduced pressure to yield alcohol **2** (257 mg, 90%) as a pale white solid. R*_f_* 0.26, (hexane/ethyl acetate v/v 1:1); mp 78-79˚C (lit.,^6^ mp 75-78˚C); ^1^H NMR: δ_H_ (CDCl_3,_ 400MHz) 3.77 (br s, 1 H, O*H*), 3.97 (s, 3 H, C*H*_3_), 4.85 (s, 2H, C*H*_2_), 7.38 (d, *^3^J* = 8.0 Hz, 1H, py 3-*H*), 8.30 (dd, *^3^J* = 8.0 Hz and *^4^J* = 3.0 Hz, 1H, py 4-*H*) and 9.17 (d, *^4^J* = 3.0 Hz, 1H, py 6-*H*); ^13^C NMR: δ_C_ (CDCl_3,_ 100.7MHz) 52.4, 64.3, 120.0, 124.9, 137.8, 149.9, 163.6 and 165.6; MS (EI^+^) *m/z* 167 (M^+^, 100); HRMS (EI^+^) calcd for C_8_H_9_NO_3_ (M^+^) 167.0582, found 167.0574 (Δ = - 5.0 ppm); Data consistent with literature.^5^

**Methyl 6-(bromomethyl)nicotinate (3).** This compound was prepared by slight modifications of a previously reported procedure.^7^ To a mixture of methyl 6-(hydroxymethyl)nicotinate (3.21 g, 19.2 mmol), tetrabromomethane (8.04 g, 24.3 mmol) and triphenylphosphine (6.37 g, 24.3 mmol) was added a minimal amount of tetrahydrofuran (30 mL). The resultant yellow solution was stirred for 2 h at room temperature; water (30 mL) was then added and the resulting mixture was extracted with dichloromethane (3 × 50 mL). The extracts were then collected and dried over sodium sulphate and concentrated under reduced pressure. The crude residue was purified by silica gel column chromatography (hexane/ethyl acetate v/v 3:2) to give the compound **3** (2.86 g, 64%) as deep red crystalline solid. R*_f_* 0.45, (hexane/ethyl acetate 3:2); mp 76-77˚C; ^1^H NMR: δ_H_ (CDCl_3,_ 400MH_z_) 3.96 (s, 3 H, C*H*_3_), 4.57 (s, 2H, C*H*_2_), 7.53 (d, *^3^J* = 8.0 Hz, 1 H, py 3-*H*), 8.28 (dd, *^3^J* = 8.0 Hz and *^4^J* = 4.0 Hz, 1H, py 4-*H*) and 9.14 (d, 1H, *^4^J* = 4.0 Hz, py 6-*H*); ^13^C NMR: δ_C_ (CDCl_3,_ 100.7MH_z_) 32.8, 52.5, 123.1, 125.2, 138.2, 150.8, 160.9 and 165.3; MS (CI^+^) *m/z* 230 (M^79^BrH^+^, 100) and 232 (M^81^BrH^+^, 98); HRMS (CI^+^) calcd for C_8_H_9_NO_2_^79^Br(M^79^Br H^+^) 229.9817, found 229.9814 (Δ = - 1.3 ppm). Data is consistent with literature.^7^

**Methyl-2-(((3-(bis(pyridin-2-ylmethyl)amino)-2-hydroxypropyl)(pyridin-2-ylmethyl)amino)methyl) isonicotinate (4).** This compound was prepared by slight modifications of a previously reported procedure.^2^ To a solution of 1-(bis(pyridin-2-ylmethyl)amino)-3-(pyridin-2-ylmethylamino)propan-2-ol (600 mg, 1.65 mmol) in dimethylformamide (5 mL) was added potassium carbonate (460 mg, 3.3 mmol) followed by the addition of a solution of methyl 6-bromomethylnicotinate (380 mg, 1.65 mmol) in DMF (5 mL). After the addition was completed, the mixture was reacted at 50˚C for 2 h. The resulting brown solution was then cooled, and poured into water, and the pH of the solution was adjusted to 8 by adding 1M hydrochloric acid. After extraction with ethyl acetate (3 × 50 mL), the extracts were collected, washed with water (100 mL) and brine (100 mL), and concentrated under reduced pressure. The residue obtained was then purified by silica gel column chromatography (dichloromethane/methanol/NH_3_(aq., 28% w/w) v/v/v 50:10:1→20:10:1), to yield compound **4** (670 mg, 79%) as a orange/brown oil. R*_f_* 0.43, (dichloromethane/methanol/NH_3_(aq., 28% w/w) v/v/v 20:10:1); ^1^H NMR: δ_H_ (CDCl_3,_ 400MH_z_) 2.65 (m, 4H, CHOHC*H_2_*), 3.88 (m, 12H, NC*H_2_*, C*H*OH, OC*H*_3_),

7.13 (m, 3H, py 5-*H*), 7.34 (m, 3H, py 3-*H*), 7.50 (d, *^3^J* = 8.0 Hz, 1H, py’ 3-*H*), 7.58 (m, 3H, py 4-*H*), 8.18 (dd, *^3^J* = 8.0 Hz and *^4^J* = 2.0 Hz, 1H, py’ 4-*H*), 8.53 (m, 3H, py 6-*H*) and 9.09 (d, *^3^J* = 2.0 Hz 1H, py’ 6-*H*); ^13^C NMR: δ_C_ (CDCl_3,_ 100.7MH_z_) 52.3, 59.0, 59.1, 60.7, 60.8, 67.2, 122.0, 122.0, 122.0, 122.5, 123.0, 136.4, 136.4, 137.4, 149.0, 149.0, 150.2, 159.3, 159.3, 164.4 and 165.8; MS (ESI^+^) *m/z* 513 (MH^+^, 100); HRMS (ESI^+^) calcd for C_29_H_33_N_6_O_6_ (MH^+^) 513.2614, found 513.2598 (Δ = - 3.1 ppm). Data is consistent with literature.^2^

**N-(2-aminoethyl)-2-(((3-(bis(pyridin-2-ylmethyl)amino)-2-hydroxypropyl) (pyridin-2-ylmethyl) amino)methyl)isonicotinamide (5).** This compound was prepared by slight modifications of a previously reported procedure.^3^ To solution methyl 2-(((3-(bis(pyridin-2-ylmethyl)amino)-2-hydroxypropyl)(pyridin-2-ylmethyl)amino)methyl)isonicotinate (600 mg, 1.10 mmol) and MeOH (5mL) was added ethylenediamine (660 mg, 11.0 mmol) at room temperature. The resulting yellow solution was stirred for 78 h and then concentrated under reduced pressure. The residue obtained was then purified by silica gel column chromatography (dichloromethane/methanol/NH_3_(aq., 28% w/w) v/v/v 50:10:2) to obtain amine **5** (499 mg, 84%) as a yellow oil. R*_f_* 0.33, (CH_2_Cl_2_:MeOH: NH_3_(aq., 28% w/w) v/v/v 50:10:2); ^1^H NMR: δ_H_ (CDCl_3,_ 400MH_z_) 2.66 (m, 4H, CHOHC*H_2_*), 3.01 (t, *^3^J* = 6.0 Hz, 2H, C*H_2_*NH_2_), 3.55 (t, *^3^J* = 6.0 Hz, 2H, NHC*H_2_*CH_2_), 3.89 (m, 9H, NC*H_2_*, C*H*OH), 7.15 (m, 3H, py 5-*H*), 7.36 (m, 3H, py 3-*H*), 7.45 (d, *^3^J* = 8.0 Hz, 1H, py’ 3-*H*), 7.61 (m, 4H, py 4-*H,* N*H*CH_2_CH_2_), 8.01 (dd, *^3^J* = 8.0 Hz and *^4^J* = 1.5 Hz, 1H, py’ 4-*H*), 8.52 (m, 3H, py 6-*H*) and 8.94 (d, *^4^J* = 1.5 Hz, 1H, py’ 6-*H*); ^13^C NMR: δ_C_ (CDCl_3,_ 100.7MH_z_) 41.0, 42.0, 59.0, 59.1, 60.6, 60.7, 61.0, 67.2, 122.0, 122.1, 122.7, 123.1, 123.1, 128.6, 135.6, 136.5, 147.4, 149.0, 149.0, 159.2, 159.3, 162.7 and 165.9; MS (ESI^+^) *m/z* 513 (MH^+^, 100); HRMS (ESI^+^) calcd for C_30_H_37_N_8_O_2_ (MH^+^) 541.3039, found 541.3031 (Δ = - 1.5 ppm). Data is consistent with literature.^3^

**N-(2-acrylamidoethyl)-2-(((3-(bis(pyridin-2-ylmethyl)amino)-2-hydroxypropyl)(pyridin-2-ylmethyl) amino)methyl)isonicotinamide (6).** This compound was prepared by slight modifications of a previously reported procedure.^4^ A dichloromethane (5 mL) solution of 1-ethyl-3-(3-dimethylaminopropyl)carbodiimide hydrochloride (92 mg, 0.48 mmol) was added dropwise to a solution of N-(2-aminoethyl)-2-(((3-(bis(pyridin-2-ylmethyl)amino)-2-hydroxypropyl)(pyridin-2-ylmethyl)amino)methyl)isonicotinamide (210 mg, 0.400 mmol), acrylic acid (35 mg, 0.48 mmol), and 4-methoxyphenol (0.5 mg, 0.004 mmol ) in dichloromethane (15 mL) at 0 °C for 5 min. The reaction mixture was stirred for 3 h at room temperature under a nitrogen atmosphere and concentrated under reduced pressure. The residue was then dissolved in chloroform (100 mL) and washed with HEPES-NaOH buffer (0.5 M, pH 7.8, 50 ml × 5) and concentrated under reduced pressure. The residue obtained was purified by silica gel column chromatography (chloroform/methanol/NH_3_(aq., 28% w/w) v/v/v 50:10:1) to obtain the acrylamide-PhosTag^TM^ compound **6** (185 mg, 78 %) as a pale yellow oil. R*_f_* 0.71, (acetonitrile/methanol: NH_3_(aq., 28% w/w) v/v/v 70:10:1); ^1^H NMR: δ_H_ (CDCl_3,_ 400MH_z_) 2.66 (m, 4H, CHOHC*H_2_*), 3.61 (m, 4H, C*H_2_*NH_2,_ C*H_2_*CH_2_), 3.87 (m, 9H, NC*H_2_*, C*H*OH), 5.65 (d, 1H, ^3^*J_cis_ =* = 10.5 Hz, CHC*H*_2_), 6.14 (dd, 1H, ^3^*J_trans_ =* 17.0 and ^3^*J_cis_ =* 10.5 Hz, C*HC*H_2_), 6.29 (d, 1H, ^3^*J_trans_* = 17.0 Hz, CHC*H*_2_), 6.92 (br s, 1H, CHCON*H*), 7.14 (dd, 3H, ^3^*J* = 10.0 and 10.5 Hz, py 5-*H*), 7.36 (d, 3H, ^3^*J* = 12.0 Hz, py 3-*H*), 7.44 (d, 1H, ^3^*J* = 8.0 Hz, py’ 3-*H*), 7.60 (dd, 3H, ^3^*J* = 12.0 and 10.0 Hz, py 4-*H*), 7.93 (br s, 1H, pyCON*H*), 8.02 (dd, 1H, ^3^*J* = 8.0 and ^4^*J* = 2.0 Hz, py’ 4-*H*), 8.50 (d, 3H, ^3^*J* = 10.5 Hz, py 6-*H*) and 8.93 (d, 1H, ^4^*J* = 2.0 Hz, py’ 6-*H*); ^13^C NMR: δ_C_ (CDCl_3,_ 100.7MH_z_) 39.9, 41.3, 59.0, 59.0, 60.6, 60.7, 67.2, 122.1, 122.7, 123.1, 127.1, 128.1, 130.4, 135.4, 136.5, 136.5, 147.7, 148.9, 149.0, 159.1, 159.3, 162.8, 166.5 and 167.2; MS (ESI^+^) *m/z* 617 (MNa^+^, 100), 595 (MH^+^, 21); HRMS (ESI^+^) calcd for C_33_H_39_N_8_O_3_ (MH^+^) 595.3145, found 595.3132 (Δ = - 2.2 ppm). Data is consistent with literature.^4^

**References**

1. L. M. Elphick, S. E. Lee, E. S. Child, A. Prasad, C. Pignocchi, S. Thibaudeau, A. A. Anderson, L. Bonnac, V. Gouverneur and D. J. Mann, *ChemBioChem*, 2009, **10**, 1519-1526.

2. *Application: EP Pat.*, 1455189, 2004.

3. E. Kinoshita, E. Kinoshita-Kikuta, K. Takiyama and T. Koike, *J. Sep. Sci.*, 2005, **28**, 155-162.

4. E. Kinoshita, E. Kinoshita-Kikuta, K. Takiyama and T. Koike, *Mol. Cell Proteomics*, 2006, **5**, 749-757.

5. H. Chong, S. V. Torti, R. Ma, F. M. Torti and M. W. Brechbiel, *J. Med. Chem.*, 2004, **47**, 5230-5234.

6. K. Pfleger, W. Fuchs and M. Pailer, *Mon. Chem.*, 1978, **109**, 597-602.

7. M. Yamaguchi, H. Kousaka, S. Izawa, Y. Ichii, T. Kumano, D. Masui and T. Yamagishi, *Inorg. Chem.*, 2006, **45**, 8342-8354
